# Activation of peroxisome proliferator–activated receptor gamma induces anti-inflammatory properties in the chicken free avian respiratory macrophages

**DOI:** 10.1186/s40781-015-0073-1

**Published:** 2015-11-20

**Authors:** Mbuvi P. Mutua, Lucilla Steinaa, Muya M. Shadrack, Gicheru M. Muita

**Affiliations:** Department of Zoological Sciences, Kenyatta University, P.O Box 43844–00100, Nairobi, Kenya; International Livestock Research Institute, P.O Box 30709–00100, Nairobi, Kenya; Department of Zoology, Jomo Kenyatta University of Agriculture and Technology, P.O Box 62000–0200, Nairobi, Kenya

**Keywords:** Avian, Free avian respiratory macrophages, Peroxisome proliferator-activated receptor, Troglitazone

## Abstract

**Background:**

Activation of peroxisome proliferator activated receptor gamma (PPAR γ) in the alveolar macrophages (AM) by selective synthetic PPAR γ ligands, improves the ability of the cells to resolve inflammation. In birds, respiratory macrophages are known as free avian respiratory macrophages (FARM) and show distinct functional differences from AM. The effects of treating FARM with PPAR γ ligands are unclear.

**Methods:**

FARM were harvested by lavage of chicken respiratory tract and their morphology assessed at microscopic level. The effects of PPAR γ agonists on the FARM in vitro viability, phagocytic capacity and proinflammatory cytokine (TNF-α) production were assessed.

**Results:**

FARM had eccentric nucleus and plasma membrane ruffled with filopodial extensions. Ultrastructurally, numerous vesicular bodies presumed to be lysosomes were present. FARM treated with troglitazone, a selective PPAR γ agonist, had similar in vitro viability with untreated FARM. However, treated FARM co-cultured with polystyrene particles, internalized more particles with a mean volume density of 41 % compared to that of untreated FARM of 21 %. Further, treated FARM significantly decreased LPS-induced TNF-α production in a dose dependent manner.

**Conclusion:**

Results from this study show that PPAR γ synthetic ligands enhance phagocytic ability of FARM. Further the ligands attenuate production of proinflammatory cytokines in the FARM, suggesting potential therapeutic application of PPAR γ ligands in the management of respiratory inflammatory disorders in the poultry industry.

## Background

Alveolar macrophages (AM) in mammals constitute first line of pulmonary defense where they expunge deposited foreign particles and kill pathogens that land on the vast and thin gas-blood tissue barrier [1]. Following infections, activated AM produce proinflammatory cytokines and other mediators of inflammation that serve to localize and remove injurious stimuli [2]. However, prolonged inflammation is maladaptive and is characterized by persistent production of proinflammatory cytokines augmenting respiratory epithelial tissue damage [3].

Peroxisome proliferator activated receptors (PPAR) are ligand activated transcription factors and three isoforms, PPAR α, PPAR δ and PPAR γ have been described [4]. The PPAR show distinct tissue distribution [5, 6] with PPAR γ being predominantly expressed in adipose tissue where it plays an important role in glucose metabolism and adipogenesis [7]. Expression of PPAR γ protein has also been demonstrated in monocytes and macrophages [8]. Thiazolidinediones are selective synthetic PPAR γ agonists [9, 10] which improve the ability of AM to restore alveolar architecture through non phlogistic clearance of inflammatory sites in the mammalian lung [11, 12]. Chicken peroxisome proliferator activated receptor gamma (chPPAR γ) is structurally different from the mammalian PPAR γ suggesting different functional roles [13, 14].

Respiratory disease conditions, partly characterized by chronic inflammation of the respiratory epithelia, cause immense economic losses in the poultry industry [15, 16]. Despite the losses, relatively little is known about the avian pulmonary cellular defense mechanisms [17, 18]. In birds, respiratory macrophages are referred to as free avian respiratory macrophages (FARM) [19, 20] and dearth of the cells in the lung air sac system has been purported to foreordain a weak innate immunity thus predisposing birds to respiratory inflictions [21–23]. However, FARM exhibit a significantly higher phagocytic ability than AM [24] and mobilization of the cells in the avian respiratory system does not occur after intravenous application of lipopolysaccharide, incomplete freunds adjuvant or glucan, compounds known to induce migration of AM from the lung interstitium into the alveolar space [25].

The effects of PPAR γ agonists on FARM are unknown. The aim of this study was, therefore, to determine:

(i) The effect of selective synthetic PPAR γ ligands on the phagocytic capacity of FARM
(ii) The effect of the PPAR γ ligands on proinflammatory cytokine production by assessing TNF-α secretion in lipopolysaccharide activated FARM.

## Methods

### Pulmonary lavage of the avian respiratory system

All experimental procedures were approved by the Kenyatta University Animal Ethics Committee. FARM were obtained from the respiratory system of mature specimens of domestic fowl as previously described [26]. Briefly, chickens were anesthetized and then euthanized by intravenous injection of an overdose of pentobarbitone sodium (Euthanase®) into the brachial vein. The trachea was then exposed and sterile pre-warmed (40 °C) phosphate buffered saline (PBS) was poured down the respiratory system. Recovered lavage fluid was centrifuged and the pelleted FARM re-suspended in sterile cell-culture medium.

### Processing of FARM for transmission electron microscopy (TEM)

Recovered FARM were fixed in 2.5 % phosphate buffered glutaraldehyde solution for 12 h. The cells were then post fixed in 1 % osmium tetraoxide in 0.1 M sodium cacodylate buffer followed by dehydration in graded replacement of ethanol (70 %, 80 %, 90 %, and 100 % twice). Gradual replacement of ethanol with propylene oxide was then done before infiltrating and embedding the cells in epoxy resin. Using Reichter® ultra-microtome, semithin and ultrathin sections were obtained from processed blocks. The semithin sections were collected on glass slides and stained with 3 % toluidine blue while the ultrathin sections were picked on copper grids, stained with uranyl acetate and lead citrate, and observed with a Philips 201C TEM under an accelerating voltage of 60 Kv. Micrographs were developed from the processed sections for morphological studies.

### In vitro viability of the FARM

FARM were washed three times in PBS and re-suspended at a concentration of 1.5 × 10^5^ cells/ml in sterile eppendorf tubes containing RPMI-1640 cell culture medium and treated with 9 μM of troglitazone (Abcam, Science Park Cambridge, UK) for 1 h. A control pellet of FARM was processed in a similar manner but without troglitazone. The tubes were kept for 4 h in an incubator (40 °C and 5 % CO_2_). A viable count of FARM was assessed using trypan blue in hemocytometer.

### Phagocytosis assays

Recovered FARM were re-suspended at concentrations of 1.5 × 10^5^ cells/ml in fresh RPMI - 1640 in sterile eppendorf tubes. The FARM were treated with 9 μM of troglitazone in incubator for 1 h. Treated and untreated FARM were co-cultured with polystyrene particles (Sigma 3050 Spruce Street, USA) in incubator for 3 h. Shaking of tubes was done regularly to ensure contact of the cells and the particles. Thereafter, FARM were fixed in 2.5 % phosphate buffered glutaraldehyde solution for 12 h and processed for TEM. Semithin and ultrathin sections were processed for estimation of diameter of the FARM and volume density of internalized particles in the cells respectively.

### Estimation of the diameters of the FARM and the volume density of the phagocytized particles

Diameters of FARM were determined under an ocular graticule with a linear scale at a magnification of × 100. In each field, to avoid bias, only diameters of FARM at the four corners of the fields were measured. The volume density of the phagocytized particles in the FARM was estimated as previously described [27]. Briefly, ultrathin sections were processed and the corresponding micrographs recorded on a 35-mm electron microscope film prior to being projected onto a screen at a final magnification of × 14 000. A quadratic lattice grid was superimposed at a random position onto each projected image. The total number of points falling onto profiles of the phagocytized particles [P (p)] and on entire cell [P (c)] was counted. Volume density of phagocytized particles [V V (p, c)] was then calculated as follows: V V (p,c) = P (p) / P (c)

### Measurement of TNF-α production by the FARM

The FARM were washed three times in PBS and seeded at a density of 1.5 × 10^5^ cells/well in RPMI 1640 with 5 % FCS into 24-well tissue culture. The cells were treated with varying doses (3 μM, 6 μM and 9 μM) of troglitazone for 1 h before addition of 0.1 ng/ml lipopolysaccharide (LPS). After 24 h incubation at 40 °C in 5 % CO_2_, the supernatants were harvested for TNF-α measurement using ELISA kit (Bicom Biotech, SA). Briefly, the supernatants were diluted appropriately and incubated with anti-chicken TNF-α antibody coated plate at 40 °C for 1 h. The plate was washed 3 times in phosphate buffered saline-tween (PBS-T) followed by addition of biotin–streptavidin HRP labeled anti-chicken TNF-α. The plate was incubated for 30 min at 40 °C followed by 3 washes in PBS-T before addition of chromogen.

### Data analysis

For paired experiments, student *t*–test was used to compare the values on the chicken FARM in the various experiments while analysis of group data for multiple comparisons was performed using ANOVA followed by Duncan’s multiple range test to determine the level of differences. The level of significance was set at *p* ≤ 0.05 confidence level. The results were presented in form of tables, graphs and micrographs. Means ± Standard Error of the Mean (SEM) were used to explain the results in text and tables.

## Results

### Morphological observations

**Fig. 1:**
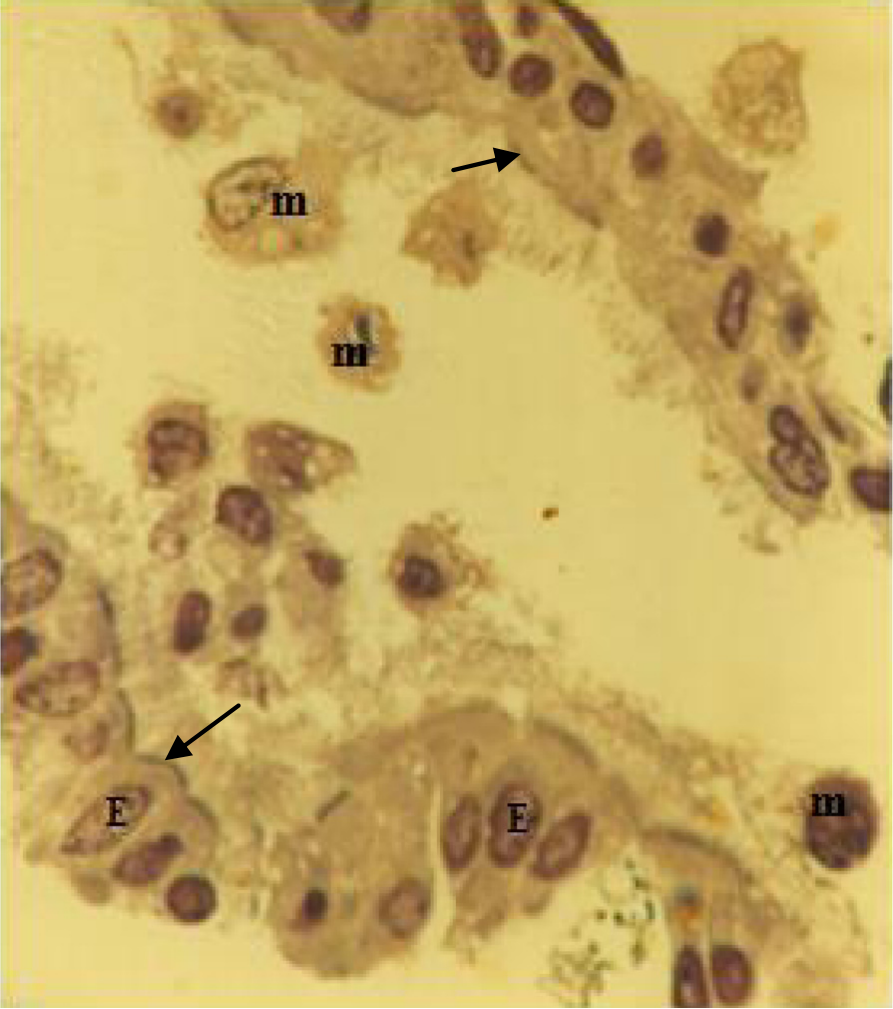
A Photomicrograph showing epithelial cells (E) with cilia (arrows) and a collection of FARM (m) recovered by lavage of chicken respiratory system. The photomicrograph was developed from semithin (1 μm) section cut from a processed block of epoxy resin embedded cell suspension. (×400)

**Fig. 2:**
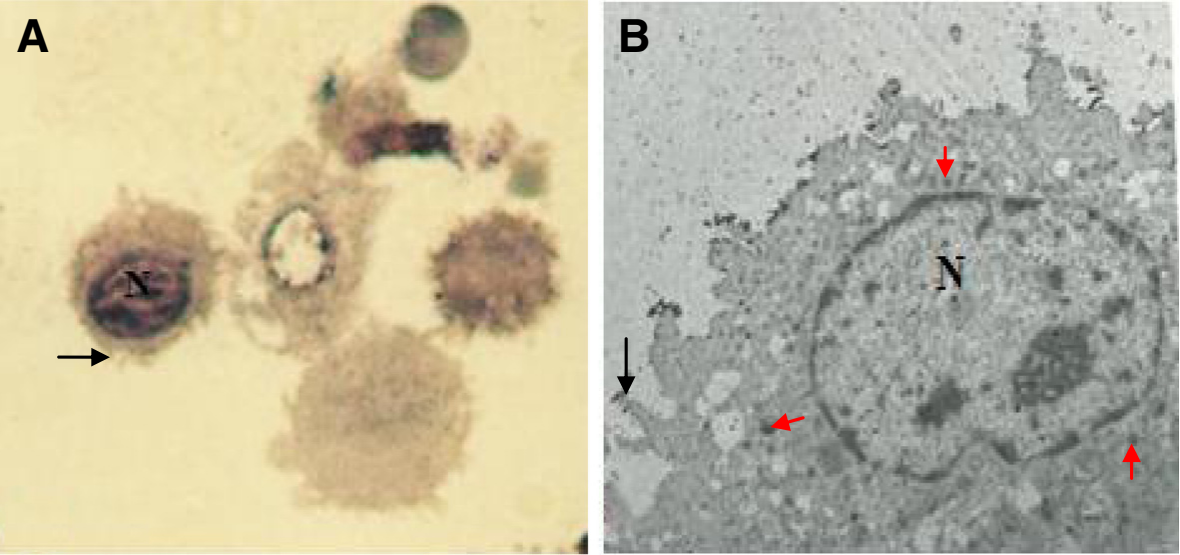
**a** Photomicrograph (×400) and (**b**) electron micrograph (×950) of FARM recovered by lavage of the chicken respiratory system. The cells have eccentric nucleus (N) and plasma membrane ruffled with filopodial extensions (arrows). In (**b**), numerous cytoplasmic vesicular bodies presumed to be lysosomes (*red arrows*) were identified. The Semithin (1 μm) and the ultrathin (80 nm) sections were cut from processed blocks of epoxy resin embedded cell suspensions

The lavage fluid recovered from the respiratory system of the chicken contained both ciliated epithelial cells and FARM (Fig. 1). Typically, the FARM had plasma membrane ruffled with filopodial extensions and an eccentrically located nucleus (Fig. 2). Ultra structurally, the FARM had variably electron dense vesicular cytoplasmic organelles presumed to be lysosomes (Fig. 2b)

### In vitro viability of the FARM

**Fig. 3:**
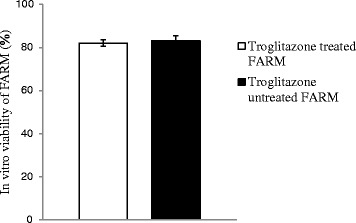
Under similar experimental conditions, there was no significant (*p* ≥ 0.05) difference in the in vitro viability of troglitazone treated and untreated FARM. The bars show SEM

Troglitazone treatment of chicken FARM at a dosage of 9 μM for 4 h did not compromise FARM viability. Troglitazone treated FARM exhibited equivalent (*p* ≥ 0.05) in vitro viability with untreated FARM under similar experimental conditions. The in vitro viability of troglitazone treated FARM and untreated FARM was 82 ± 1.5 % and 83 ± 2.5 % respectively (Fig. 3)

.

### Morphometric observations

**Fig. 4:**
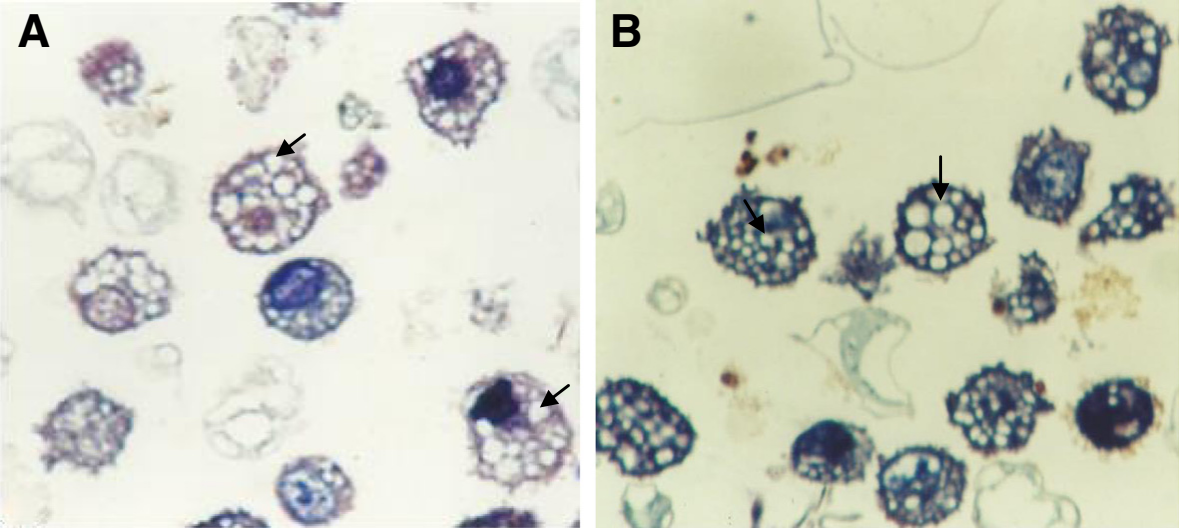
Photomicrographs showing internalized polystyrene based particles (arrows) in (**a**) troglitazone untreated FARM and (**b**) troglitazone treated FARM (**b**). The FARM and the particles were co-cultured in RPMI 1640 culture medium for 3 h. The photomicrographs were prepared from semithin (1 μm) sections cut from processed blocks of epoxy resin embedded cell suspension. (×400)

**Fig. 5:**
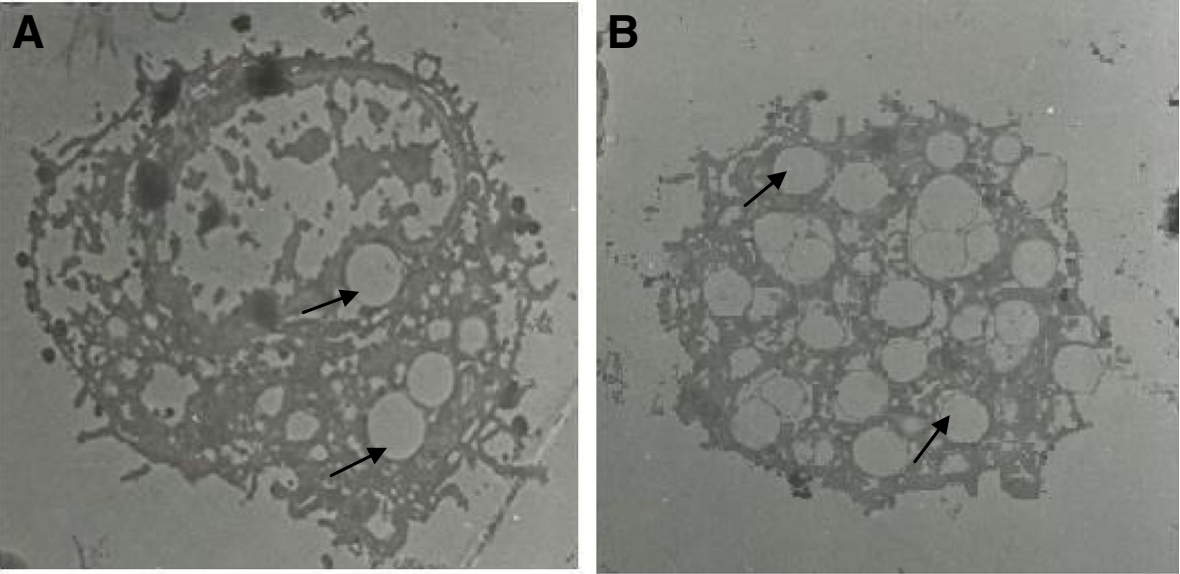
Electron micrographs showing internalized polystyrene particles (arrows) in (**a**) troglitazone untreated and (**b**) troglitazone treated chciken FARM. Three and twenty partciles have been phagocytozed in (**a**) and (**b**) respectively. In (**b**) the particles are engulfed in irregular vacoules presumably an indication of enhanced destruction of internalized particles. The micropraphs were developed from ultrathin (80 nm) sections cut from blocks of epoxy resin embedded cell suspension. (×950)

**Fig. 6:**
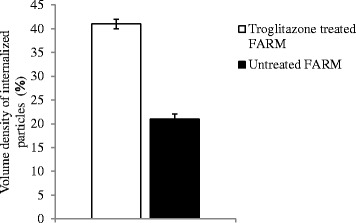
Comparison of the mean volume density of internalized particles in troglitazone treated FARM and in untreated chicken FARM. Troglitazone treated FARM significantly (*p* ≤ 0.05) internalized more polystyrene particles than untreated FARM. Bars show SEM

The mean diameter of troglitazone treated FARM was not significantly (*P* ≥ 0.05) different from that of untreated FARM (Table 1). Quantitative estimation of loading of FARM with polystyrene particles was assessed using micrographs (Figs. 4 and 5). Despite having equivalent diameters and therefore volume, the mean volume density of internalized particles per unit volume of treated FARM was 41 ± 1.0 %, a significant (*P* ≤ 0.05) value compared to that of the untreated FARM which was 21 ± 1.1 % (Fig. 6).

**Table 1:** Mean diameter of troglitazone treated and untreated FARM

Slide Number	Diameter of treated FARM (μm)	Diameter of untreated FARM (μm)
1	12	14
2	15	10
3	12	11
4	11	12
5	13	12
6	10	10
7	11	13
8	9	13
9	14	11
10	12	12
11	10	11
12	12	13
Mean	11.7	11.8
SEM	0.5	0.4

Data represent the mean diameter of troglitazone treated and untreated FARM that were co-cultured with polystyrene particles. The mean diameter of treated FARM was not significantly (*p* ≥ 0.05) different from that of untreated FARM

### The effect of troglitazone on TNF- α production by the chicken FARM

**Fig. 7:**
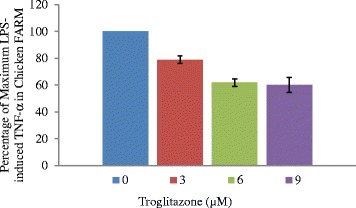
Comparison of TNF-α production by LPS-activated chicken FARM treated with varying doses of troglitazone. Treatment of FARM with increasing doses of troglitazone significantly (*p* ≤ 0.05) inhibited TNF-α production by the FARM. The bars show SEM

To define the functional role of PPAR-γ in the chicken FARM, the effect of troglitazone on cytokine production by the chicken FARM was measured by determining TNF-α concentrations in culture supernatants of lipopolysaccharide-stimulated FARM after treatment with graded (3 μM, 6 μM and 9 μM) doses of troglitazone. Lipopolysaccharide elicited considerable amounts of TNF-α production by FARM at concentration of 0.1 ng/ml. Addition of troglitazone to cultures of LPS-induced chicken FARM, significantly (*p* ≤ 0.05) inhibited TNF-α production by the FARM in a dose dependent manner (Fig. 7).

## Discussion

In the present study, we examined the effect of PPAR γ ligands on the phagocytic capacity of FARM. This study reports for the first time that the phagocytic capacity of freshly harvested chicken FARM is enhanced by selective synthetic PPAR γ ligands. Selective synthetic PPAR γ ligands improve the phagocytic ability of AM with subsequent clearance of inflammatory site, an essential process during restoration of alveolar architecture in the mammalian lung [28]. Chronic inflammation, partly characterized by accumulation of FARM with diminished phagocytic ability in the inflammatory site, causes gross respiratory epithelial tissue destruction with subsequent high mortality in the poultry industry [29, 30]. Phagocytosis is the most important defense mechanism in all phyla of the animal kingdom [31] and therefore, up regulation of phagocytic ability of FARM by PPAR γ agonists could be critical in clearance of inflammatory stimuli in the avian lung. In this study, PPAR γ ligands substantially improved the phagocytic ability FARM. Further, troglitazone treated FARM had irregular vacuoles formed around ingested particles indicating up regulated destruction of internalized particles by the FARM.

A characteristic of non phlogistic phagocytosis is the ability of activated macrophages to clear inflammatory stimuli with diminished production of proinflammatory cytokines [32]. To elucidate the non phlogistic functional role of PPAR γ ligands during phagocytosis in the FARM, we treated lipopolysaccharide activated FARM with varying doses of troglitazone. Troglitazone treated FARM inhibited TNF-α production in lipopolysaccharide activated FARM in a dose dependent manner. TNF-α has been reported as the primary regulator of inflammation [33] and activated FARM produce TNF-α in response to respiratory inflictions [34]. However, incessant production of proinflammatory cytokines prolongs inflammation contributing to pathogenesis of respiratory disease conditions such as aspergillosis [35]. An understanding of the mechanisms that enhance FARM to regulate inflammatory responses may permit development of products for the enhancement productivity in the poultry industry. FARM are the predominant immune cells in the avian lung [36] therefore, synthetic PPAR γ agonists could be used in attenuating proinflammatory cytokine production by the cells as a therapeutic intervention in resolving respiratory inflammatory disease conditions in the poultry industry.

## Conclusion

In this study, selective synthetic PPAR γ agonists significantly enhanced the phagocytic index of chicken FARM. Further, the PPAR γ ligands attenuated production of proinflammatory cytokine TNF-α by activated FARM. This study, therefore, concludes that PPAR γ ligands are attractive therapeutic novel drug targets for resolution of avian respiratory inflammatory disease conditions.
